# Prematurity, asphyxia and congenital malformations underrepresented among neonates in a tertiary pediatric hospital in Vietnam

**DOI:** 10.1186/1471-2431-12-199

**Published:** 2012-12-29

**Authors:** Alexandra Y Kruse, Binh TT Ho, Cam N Phuong, Lone G Stensballe, Gorm Greisen, Freddy K Pedersen

**Affiliations:** 1 International Child Health Research Unit, JMC, Rigshospitalet – Copenhagen University Hospital, Copenhagen, Denmark; 2 Neonatal Intensive Care Unit, Children’s Hospital 1, Ho Chi Minh City, Vietnam; 3 Department of Pediatrics and Adolescent Medicine, JMC, Rigshospitalet – Copenhagen University Hospital, Copenhagen, Denmark; 4 Department of Neonatology, JMC, Rigshospitalet – Copenhagen University Hospital, Copenhagen, Denmark; 5 Faculty of Health and Medical Science, University of Copenhagen, Copenhagen, Denmark

**Keywords:** Developing country, Health care access, Hospitalization, Lower middle income country, morbidity, Mortality, Neonate, Newborn, Vietnam

## Abstract

**Background:**

Estimated 17,000 neonates (≤ 28 days of age) die in Vietnam annually, corresponding to more than half of the child mortality burden. However, current knowledge about these neonates is limited. Prematurity, asphyxia and congenital malformations are major causes of death in neonates worldwide. To improve survival and long term development, these vulnerable neonates need access to the specialized neonatal care existing, although limited, in lower middle-income countries like Vietnam. The aim of this study was to describe these conditions in a specialized Vietnamese hospital, compared to a Danish hospital.

**Methods:**

We performed a comparative observational study of all neonates admitted to a tertiary pediatric hospital in South Vietnam in 2009–2010. The data were prospectively extracted from the central hospital registry and included basic patient characteristics and diagnoses (International Classification of Diseases, 10^th^ revision). Prematurity, asphyxia and designated congenital malformations (oesophageal atresia, gastroschisis, omphalocoele, diaphragmatic hernia and heart disease) were investigated. In a subgroup, the prematurity diagnosis was validated using a questionnaire. The hospitalization ratio of each diagnosis was compared to those obtained from a Danish tertiary hospital. The Danish data were retrieved from the neonatal department database for a ten-year period.

**Results:**

The study included 5763 neonates (missing<1%). The catchment population was 726,578 live births. The diagnosis was prematurity in 7%, asphyxia in 2% and one of the designated congenital malformations in 6%. The diagnosis of prematurity was correctly assigned to 85% of the neonates, who were very premature or had very low birth weight according to the questionnaire, completed by 2196 neonates. Compared to the Danish Hospital, the hospitalization ratios of neonates diagnosed with prematurity (p<0.01), asphyxia (p<0.01) and designated congenital malformations (p<0.01- 0.04) were significantly lower.

**Conclusion:**

Our findings suggest the investigated diagnoses were underrepresented in the Vietnamese study hospital. In contrast, relatively mild diagnoses were frequent. These results indicate the use of specialized care may not be optimal. Pre-hospital selection mechanisms were not investigated and additional studies are needed to optimise utilisation of specialized care and improve neonatal survival.

## Background

The vast majority of the millions of children dying before the age of 5 are born in developing countries; neonates (≤ 28 days of age) constitute more than 40% of these deaths. Efforts to reduce neonatal mortality have lagged behind, hampering fulfilment of the millennium development goal to reduce child mortality. The major causes of neonatal mortality globally are infections (36%), prematurity (28%), asphyxia (23%) and congenital malformations (7%) [1-4]. Prematurity is an underlying cause in half of the neonatal deaths. Gestational age (GA) is often unknown, and birth weight (BW) is used as a proxy for maturity. Very premature neonates (VP, GA<32 weeks) and very low birth weight (VLBW, BW ≤ 1500 g) neonates have a particularly high risk of death. These neonates represent approximately one quarter of all premature neonates [5].

Vietnam has risen from one of the poorest countries in the world to a lower middle-income country, like India or Ghana. With the emerging economy, health care has improved and child mortality has declined, but less convincingly for neonates [3,6,7]. The neonatal mortality rate (NMR) of 12/1000 live births in Vietnam represents more than half of the total child mortality. By comparison, the NMRs of Western Europe are among the lowest in the world (1-4/1000 live births), and the NMRs of Sub-Saharan Africa are among the highest (45-50/1000 live births) [8].

In Vietnam, an estimated 17,000 neonates die annually [3]. Data on neonatal morbidity and mortality are scarce, however, and most figures rely on estimates and models. There is a paucity of peer–reviewed neonatal hospital studies, even though most neonatal deaths are anticipated to occur in hospitals. Almost 90% of women deliver in health care facilities [9,10] and presumably remain hospitalized the first days after delivery, which is the most vulnerable period for neonates [1].

If we want to improve the survival and later development of vulnerable groups of neonates, we believe they need access to specialized care. However, specialized neonatal care is limited in Vietnam, with only basic and intermediary neonatal care available in the vast majority of the provinces [11]. We explored the access to existing specialized care. We hypothesised that neonates with prematurity, asphyxia and designated congenital malformations were underrepresented in the study hospital providing specialized neonatal care.

We investigated these conditions, for the following reasons: they are major causes of neonatal mortality globally, their diagnostic criteria are relatively well-defined, they are fairly easy to diagnose and their incidences are roughly comparable [5,12-14]. Infections did not meet these criteria and were therefore not investigated. We compared to a similar hospital in Denmark, where we assumed there would be less selection bias in admissions.

The present study describes the hospitalization ratios of these conditions in a tertiary pediatric hospital in Vietnam. The ratios are compared to those of a Danish tertiary hospital and to estimated incidences within the catchment population.

## Methods

### Setting

The present study was conducted at Pediatric Hospital Number 1 in Ho Chi Minh City (PH1). This hospital is a tertiary referral hospital for South Vietnam (population 42 million). It has 1,200 beds and receives 86,000 children (2/3 are from the provinces and 1/3 are from Ho Chi Minh City). Approximately 95% of the patients are referred from other health care facilities. The neonatal department has 150 beds and includes basic, semi-intensive and intensive care units. The specialized neonatal care available in the study period included exchange transfusion, surfactant replacement, ventilator support including high frequency ventilation, and surgery. In 2009 the potential catchment population comprised 726,578 live births in South Vietnam (approximately half of the deliveries in the country) [15]. The sex ratio at birth was 109.7 (boys/100 girls) [16].

PH1 provides the most specialized neonatal care in the country and is responsible for organising neonatal care in the southern part of Vietnam. Specialized neonatal care in the South was provided by four tertiary hospitals, all situated in Ho Chi Minh City: two pediatric hospitals and two maternity hospitals. In 2009, the other pediatric hospital admitted 3,252 neonates (11% birth weight < 2500 g) and had no neonatal intensive care unit. The two maternity hospitals performed 67,655 deliveries (more than 95% of the deliveries registered in the city). Of these, 18,328 were admitted to their neonatal units, including the neonatal intensive care units. No neonatal surgery was offered in the maternity hospitals.

### Patients

All neonates admitted to PH1 in a 12 month period from February 2009 to February 2010 were included in the study. The neonates were identified using the central hospital registry.

For a subgroup of neonates a questionnaire was completed. The subgroup included all of the neonates in emergency room and intensive care unit as well as every second neonate from the semi-intensive care unit, whereas neonates in the basic care unit were not included.

### Ethics

In Vietnam, the study was approved by The Scientific Review Board and Ethical Committee of the study hospital. In Denmark, The Danish Data Protection Agency approved the study. The hospital department approved the use of the Danish database. This study was exempt from ethical approval, since observational studies do not constitute a health research project according to The Danish National Committee on Health Research Ethics.

### Data collection

The admitting nurse enters data into the central hospital registry for all. Name, file number, sex, birth date, and admission date are recorded. At discharge, the discharging nurse enters the date and the International Classification of Diseases 10^th^ revision (ICD-10) diagnoses assigned by the doctor. If more diagnoses are relevant, the doctors are instructed to assign the most important diagnoses including the underlying disease diagnosis.

In the study, only the first hospitalization was included, if admitted more than once within the neonatal period. The data were obtained prospectively, and the final data extraction was conducted six months after including the last patient (to ensure complete discharge data).

For a subgroup GA and BW were obtained from a questionnaire completed at admission by the doctor receiving the patient.

### Diagnoses investigated

Besides the ICD-10 diagnoses described above, relatively mild diagnoses were defined and registered. They were defined as diagnoses, which could probably be adequately managed by lower levels of care.

### Diagnosis validity

The diagnosis of prematurity was validated using the GA or BW in the subgroup questionnaire (n=2196). GA was preferred, if GA unknown BW was used. We focused on VP or VLBW. If the neonate was VP or VLBW, assignment of prematurity diagnosis was considered correct.

### Hospitalization ratios compared to a Danish Hospital

We compared the hospitalization ratios (number of hospital admissions/1000 live births in the catchment area) in PH1 to those in a general hospital providing tertiary neonatal care in Copenhagen in Denmark (Rigshospitalet, RH)

In RH, the ICD-10 diagnoses, sex, GA and BW were obtained from the neonatal department database for 2001–2010, and annual means were calculated. The mean of a 10-year period was chosen due to the smaller population in Denmark and hence the lower hospitalization numbers. The discharging doctor completes a separate database record on paper for each patient, which is checked by a consulting doctor before a secretary enters the data into the database. The doctors are instructed to assign all relevant diagnoses.

RH serves as local hospital to a part of Copenhagen and as tertiary general hospital for East Denmark. Obstetric care (including centralisation of deliveries with GA<28 weeks) and neonatal care are among the services provided. The neonatal department, including intensive care, has 36 beds and manages both neonates born in the hospital and those referred from other hospitals. Six of the referring hospitals provide specialized care. None of these offer surgery in neonates and only one has ventilator capacity, which is limited. In addition to the therapies offered at PH1, RH provided inhaled nitric oxide, extracorporeal membrane oxygenation, extensive surgical procedures and in the end of the study period controlled hypothermia. In 2009, the catchment population was 29,161 live births [17], corresponding to 1/25 of the PH1 catchment population. The birth sex ratio was 105 [8].

### Data analyses

The data were entered into Microsoft Access version 97 and analysed using STATA IC, version 11 (Texas, USA). Patients were excluded from the analysis if date of birth, admission date or diagnosis were missing. The data are reported as medians, 25 – 75% interquartile ranges and ratios. Comparisons were conducted using Chi-square test. Two sided p-values were calculated, and the significance level was set at 5%.

## Results

### Patients

In total, 5802 neonates were admitted during the study period, corresponding to 0.8% of the catchment population of 726,578 live births. Thirty-nine (<1%) neonates were excluded from the analysis (4 due to missing dates of birth and 35 due to missing discharge dates or diagnoses). Therefore, 5763 neonates were included in the analysis.

The subgroup consisted of 2264 neonates. In 43 the questionnaire was incomplete and in 25 discharge data were missing, leaving 2196 neonates for analysis.

### Characteristics

The median admission age was 7 days (interquartile range 2–17 days) and the median length of stay was 7 days (interquartile range 4–15 days). Boys made up 55% of the population (3186/5763).

**Figure 1:**
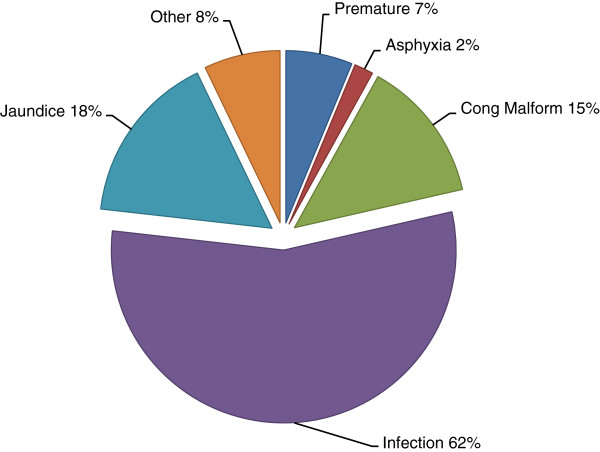
**ICD-10 diagnosis distribution among all neonates admitted (n=5763).** Two diagnoses were assigned to 780 patients (13%).

The distribution of the ICD-10 diagnoses is shown in Figure 1. Two diagnoses were assigned to 780 of the patients (14%). The major groups included infection (62%), jaundice (18%), designated congenital malformations (6%), other congenital malformations (9%), prematurity (7%) and asphyxia (2%). Relatively mild diagnoses, including sub-categories of jaundice (unspecified or physiological), infection (unspecified, viral, skin, or acute upper respiratory) and gastro-oesophageal reflux were assigned to 24% of the neonates (assigned only one diagnosis).

### Diagnosis validity

In the subgroup (n=2196 neonates), GA was unknown in 373 (7%) and BW was unrecorded in 21 (1%). Among the VP (257) or VLBW (79), 286 (85%) were correctly assigned the diagnosis of prematurity.

### Hospitalization ratios compared to a Danish hospital

Table 1 shows the hospitalization ratios of prematurity, asphyxia and designated congenital malformations in the study hospital and the Danish hospital RH. Data were available for all 8849 of the neonates admitted to RH during the study period. We found a 25 fold difference in hospitalization ratios of prematurity (0.53 versus 13.24) and asphyxia (0.17 versus 3.99) between the Vietnamese and the Danish hospitals. These where the most pronounced differences. All of the hospitalization ratios, however, were significantly lower in the study hospital (p<0.05), when taking the difference in catchment populations into account.

**Table 1:** Comparison of selected ICD-10 diagnoses in the Vietnamese and Danish Hospital

**Diagnose**	**PH1 Vietnam (n=5763)**		**RH Denmark (n=885)**		**p-value**
	**Registered**	**/1000 live births**	**Registered**	**/1000 live births**	
Prematurity	385	0.53	384	13.24	<0.01
very premature	385*	0.53	206	7.06	<0.01
**Asphyxia**	120	0.17	116	3.99	<0.01
**Cong Malformations**					
Oesophaus atresia	46	0.06	8	0.27	<0.01
Gastrochiesis	31	0.04	6	0.21	<0.01
Omphalocele	22	0.03	2	0.07	0.04
Diaphragmatic hernia	39	0.05	4	0.14	0.01
Heart Disease	222	0.31	65	2.23	<0.01

The numbers of registered diagnoses and corresponding ratios per 1000 live births in the catchment area.

PH1: Tertiary paediatric hospital in Vietnam (Paediatric Hospital No. 1) with a catchment population of 726,578 live births (2009).

RH: General tertiary hospital in Denmark (Rigshospitalet) with a catchment population of 29,161 live births (2009), annual means for the period 2001–10.

Very premature: gestational age < 32 weeks.

*Maximum estimate corresponding to all of the registered premature neonates (gestational age and birth weight is only available for a subgroup).

Compared to the sex birth ratio in the catchment populations, significantly more boys were admitted in both hospitals (p<0.01). The male admission ratios did not differ between the two hospitals (p=0.49).

## Discussion

The neonatal hospitalization ratios of prematurity, asphyxia and designated congenital malformation diagnoses were significantly lower in the study hospital in Vietnam compared to the Danish hospital. Further, the diagnoses studied were also considerably lower in the study hospital than expected from conservative catchment population estimates. By contrast, almost a quarter of the admitted patients did not seem to require specialized care.

### Diagnosis validity

The doctors assigned diagnoses according to the ICD-10 classification. The validity of the diagnosis of prematurity was fairly good. Using low BW as a proxy for prematurity is less of a problem for VLBW. We validated the prematurity diagnosis for several reasons: it was feasible; the complications of prematurity contribute to half of all neonatal deaths; and unlike the other diagnoses investigated, prematurity is not itself a disease. Therefore, it was important to investigate whether prematurity or rather later complications were assigned as diagnosis, to evaluate whether the diagnosis was a valid indicator of VP and VLBW. It is in agreement with global neonatal mortality research to use VP as a primary cause of death. The other two groups of diagnoses could not be systematically validated. It is possible that asphyxia is under-reported due to insufficient delivery information and concerns that this diagnosis may be interpreted as suboptimal delivery management. The designated congenital malformation diagnoses are likely to be more valid. The figures correspond to the annual ward reports, which are registered separately, but not traceable to individuals. Finally, the ICD-10 classification allows for comparative studies, but has limitations. The classification is widely used in research, but was created for administrative purpose. Data quality may be of concern, since it does not imply specific diagnostic criteria and errors may occur due to missing or incorrect clinical data or coding [18-20].

### Hospitalization ratios

The investigated hospitalization ratios were significantly lower compared to a Danish hospital. However, the assumption of similar incidences in the two catchment populations can be disputed. As access to antenatal ultrasound scans are likely to differ widely, abortion on medical indications are likely to differ too. Also provider-initiated preterm deliveries might differ between resource poor settings and resource rich settings. Furthermore, it is likely that pre-hospital selection mechanisms vary. Therefore, we also compared our findings to conservative catchment population incidence estimates adjusted to the context. The observed ratios are still considerably lower. The estimated incidence of VP neonates in less developed Southeast Asian countries is presumed to be more than 40 fold higher in the catchment population than registered in PH1 [21-24]. Likewise, the estimated incidence of severe asphyxia (hypoxic ischemic encephalopathy) in settings with a NMR similar to Vietnam’s is estimated to be 40 fold higher than the registered [13]. No context specific incidences are available for the designated congenital malformations. The available estimates are 3 to 20 fold higher than registered in PH1 [14,25-32], the discrepancy was most pronounced for congenital heart diseases (including all congenital heart diseases). It is possible that some of these cases may present later in infancy, which could explain part of the difference. Another possible explanation is the pre-hospital selection, due to limitations in diagnostic and management possibilities and availability of other specialized care providers.

### Population selection

The population is highly selected; it constitutes only 0.8% of the huge catchment population. All of the neonates are born elsewhere and various circumstances may influence the decision to present to the study hospital. Poor prognosis (as evaluated by staff or family), death, misdiagnosis, transportation limitations, local management, lack of treatment options in the study hospital, limited support for families of children with special needs, the 2-children policy and hidden user fees despite a policy of free pediatric admissions may all contribute to not being selected for specialized care [3,5,13].

The position and level of the two hospitals compared were similar. But the size differed. To make up for this we choose different inclusion periods. We assumed the admission rates in the Danish hospital to be less biased by admission selection for a number of reasons. In the Danish catchment area deliveries at risk of needing neonatal intensive care are referred to deliver in this hospital, the catchment area is much smaller (in square kilometres and number of live births), neonatal intensive care are provided during transport, health care is for free without user fees or incentives and includes extensive support for children with special needs, and the standard of treatment possibilities are high. Further, medical staff and families may be stronger advocates for active and full treatment of vulnerable neonates. If this is true, the Danish hospital admissions may show a more unbiased and true picture of the burden of the selected conditions, and is therefore relevant to compare to.

The sex ratio at birth were unbalanced (>107) in Vietnam and balanced in Denmark, according to United Nations Population Fund [33]. More boys were admitted in both hospitals and the ratios did not differ significantly. Therefore, perhaps surprisingly, we found no evidence of gender bias in access to specialized care in the Vietnamese hospital. It is, however, well established that male neonates are more vulnerable [1,34,35].

The neonatal bed occupancy during the study period was 154%. Almost a quarter of the patients had relatively mild diagnoses and could probably be adequately managed at lower levels of care, even though the vast majority was referred from other health care facilities. This hospitalization pattern may reflect sub-optimal utilisation of specialized neonatal care and may not to be unique for this hospital.

### Increasing access to specialized care

Even considering the other three hospitals providing specialized neonatal care, the hospitalization numbers in the study hospital were still low for the catchment population. The maternity hospitals admit only neonates delivered in their hospital, leaving 90% of the catchment population for the pediatric hospitals. Only 1.3% of this group was admitted. This ratio is 8 fold lower than the hospitalization ratios in Denmark and lower than the ratios of other developing countries [34,36,37]. However, variations in catchment populations and definition of specialized care allow only rough comparisons of trends. Probably most of the neonates missing in PH1 are cared for at lower levels.

Specialized neonatal care in the provinces, however, is limited. In 30/32 provinces in the catchment area, the highest level of neonatal care available was basic care in 10 provinces, intermediate care in 19 provinces and intensive care in 1 province (PH1). Only PH1 had the capacity of mechanical ventilation more than 24 hours and surfactant replacement [11]. Furthermore, the mean neonatal case fatality rate was more than 3 fold higher (15%) at the basic care level than at the intensive care level.

However, we do not know how Vietnamese families perceive the needs of their neonates, if born with any of the conditions investigated. They may not want their neonates to survive at any cost, considering the short and long time perspective for the family and for the neonate.

But if we want to improve survival and minimize long term deficits, these vulnerable groups need access to specialized care. This warrants access to existing specialized care and upgrading to specialized neonatal care at provincial level. This is in accordance with the current official recommendations [38], that lower levels have to upgrade and provide specialized neonatal care.

## Conclusion

This study contributes to the limited data on neonatal hospitalization in Vietnam. Our findings suggest that prematurity, asphyxia and designated congenital malformations are underrepresented in the Vietnamese study hospital. These conditions are major causes of neonatal mortality globally. In contrast, almost a quarter of the diagnoses was relatively mild. Our findings indicate that the use of specialized care may not be optimal. This is of importance to save lives and ensure cost-effectiveness, especially in resource-poor settings like Vietnam. Pre-hospital selection mechanisms were not investigated and additional studies are needed to optimise utilisation of existing care. Further, implementation of specialized neonatal care in the provinces should be prioritized to increase neonatal survival.

## Abbreviations

BW: Birth weight; GA: Gestational age; ICD-10: International Classification of Diseases, 10th revision; NMR: Neonatal Mortality Rate (deaths ≤ 28 days of age/1000 live births); PH1: Pediatric Hospital Number 1 in Ho Chi Minh City, Vietnam; RH: Rigshospitalet Copenhagen, Denmark; VP: Very premature (GA<32 weeks); VLBW: Very low birth weight (birth weight≤1500 g).

## Competing interests

The authors declare that they have no competing interests.

## Authors’ contributions

All of the authors contributed to study planning, discussion and interpretation of the data. All of the authors read and approved the final version of the manuscript. The data collection and data management were performed by HB and AK. AK performed the analysis and wrote the first draft of the manuscript.

## Pre-publication history

The pre-publication history for this paper can be accessed here:

http://www.biomedcentral.com/1471-2431/12/199/prepub

## References

[B1] Lawn JE Cousens S Zupan J 4 million neonatal deaths: when? Where? Why? Lancet 2005 365 891 900 10.1016/S0140-6736(05)71048-5 15752534

[B2] Black RE Cousens S Johnson HL Lawn JE Rudan I Bassani DG Jha P Campbell H Walker CF Cibulskis R Eisele T Liu L Mathers C Global, regional, and national causes of child mortality in 2008: a systematic analysis Lancet 2010 375 1969 1987 10.1016/S0140-6736(10)60549-1 20466419

[B3] Oestergaard MZ Inoue M Yoshida S Mahanani WR Gore FM Cousens S Lawn JE Mathers CD Neonatal mortality levels for 193 countries in 2009 with trends since 1990: a systematic analysis of progress, projections, and priorities PLoS Med 2011 8 e1001080 10.1371/journal.pmed.1001080 21918640 PMC3168874

[B4] Saugstad OD Reducing global neonatal mortality is possible Neonatology 2011 99 250 257 10.1159/000320332 21088433

[B5] Simmons LE Rubens CE Darmstadt GL Gravett MG Preventing preterm birth and neonatal mortality: exploring the epidemiology, causes, and interventions Semin Perinatol 2010 34 408 415 10.1053/j.semperi.2010.09.005 21094415

[B6] Acuin CS Khor GL Liabsuetrakul T Achadi EL Htay TT Firestone R Bhutta ZA Maternal, neonatal, and child health in southeast Asia: towards greater regional collaboration Lancet 2011 377 516 525 10.1016/S0140-6736(10)62049-1 21269675 PMC7159081

[B7] Hoa DP Nga NT Malqvist M Persson LA Persistent neonatal mortality despite improved under-five survival: a retrospective cohort study in northern Vietnam Acta Paediatr 2008 97 166 170 10.1111/j.1651-2227.2007.00604.x 18254906

[B8] The World Health Organization World Health Statistics 2011 2012 Geneva

[B9] The United Nations International Children’s Fund Vietnam Country statistics 2009 [http://www.unicef.org/infobycountry/ Vietnam_statistics.html]

[B10] General Statistics Office of Viet Nam UNICEF Viet Nam committee for Population Family and Children Viet Nam Multiple Indicator Cluster Survey 2006 – MICS 3 2007 Hanoi

[B11] Tham TTT Phuong CN Nhan NLT Investigating the level of care at newborn care units of central hopsitals in provinces and cities in Southern Vietnam by the end of year 2006 [abstract] Early Hum Dev 2008 84 41

[B12] Chao PH Huang CB Liu CA Chung MY Chen CC Chen FS Ou-Yang MC Huang HC Congenital diaphragmatic hernia in the neonatal period: review of 21 years’ experience Pediatr Neonatol 2010 51 97 102 10.1016/S1875-9572(10)60018-6 20417460

[B13] Lawn JE Lee AC Kinney M Sibley L Carlo WA Paul VK Pattinson R Darmstadt GL Two million intrapartum-related stillbirths and neonatal deaths: where, why, and what can be done? Int J Gynaecol Obstet 2009 107 Suppl 1 S5 S18 S19 19815202 10.1016/j.ijgo.2009.07.016

[B14] van der Linde D Konings EE Slager MA Witsenburg M Helbing WA Takkenberg JJ Roos-Hesselink JW Birth prevalence of congenital heart disease worldwide: a systematic review and meta-analysis J Am Coll Cardiol 2011 58 2241 2247 10.1016/j.jacc.2011.08.025 22078432

[B15] The Ministry of Health of Vietnam The Department of Reproductive Health Reproductive Health Statistics 2009 2011 Hanoi

[B16] The General Statistics Office of Viet Nam The 2009 Viet Nam Population and Housing Census, Major findings: sex ratio at birth by province/city, 2009 2010 Hanoi

[B17] Danish Board of Health Health Data 2010 [http://www.ssi.dk/Sundhedsdataogit/Registre/Fodselsregister.aspx]

[B18] De CC Quan H Finlayson A Gao M Halfon P Humphries KH Johansen H Lix LM Luthi JC Ma J Romano PS Roos L Sundararajan V Tu JV Webster G Ghali WA Identifying priorities in methodological research using ICD-9-CM and ICD-10 administrative data: report from an international consortium BMC Health Serv Res 2006 6 77 10.1186/1472-6963-6-77 16776836 PMC1513221

[B19] Ford JB Roberts CL Algert CS Bowen JR Bajuk B Henderson-Smart DJ Using hospital discharge data for determining neonatal morbidity and mortality: a validation study BMC Health Serv Res 2007 7 188 10.1186/1472-6963-7-188 18021458 PMC2216019

[B20] Vance GA Niederhauser A Chauhan SP Magann EF Dahlke JD Muraskas JK Morrison JC Does the International Classification of Disease (ICD-9) code accurately identify neonates who clinically have hypoxic-ischemic encephalopathy? Gynecol Obstet Invest 2011 71 202 206 10.1159/000318204 21160147

[B21] Beck S Wojdyla D Say L Betran AP Merialdi M Requejo JH Rubens C Menon R Van Look PF The worldwide incidence of preterm birth: a systematic review of maternal mortality and morbidity Bull World Health Organ 2010 88 31 38 10.2471/BLT.08.062554 20428351 PMC2802437

[B22] Graner S Klingberg-Allvin M Phuc HD Huong DL Krantz G Mogren I Adverse perinatal and neonatal outcomes and their determinants in rural Vietnam 1999–2005 Paediatr Perinat Epidemiol 2010 24 535 545 10.1111/j.1365-3016.2010.01135.x 20955231

[B23] Lawn JE Gravett MG Nunes TM Rubens CE Stanton C Global report on preterm birth and stillbirth (1 of 7): definitions, description of the burden and opportunities to improve data BMC Pregnancy Childbirth 2010 10 Suppl 1 S1 10.1186/1471-2393-10-S1-S1 20233382 PMC2841772

[B24] Blencowe H Cousens S Oestergaard MZ Chou D Moller AB Narwal R Adler A Vera GC Rohde S Say L Lawn JE National, regional, and worldwide estimates of preterm birth rates in the year 2010 with time trends since 1990 for selected countries: a systematic analysis and implications Lancet 2012 379 2162 2172 10.1016/S0140-6736(12)60820-4 22682464

[B25] Askarpour S Ostadian N Javaherizadeh H Chabi S Omphalocele, gastroschisis: epidemiology, survival, and mortality in Imam Khomeini hospital Ahvaz-Iran. Pol Przegl Chir 2012 84 82 85 10.2478/v10035-012-0013-4 22487740

[B26] Chen IL Lee SY Ou-Yang MC Chao PH Liu CA Chen FS Chung MY Chen CC Huang HC Clinical presentation of children with gastroschisis and small for gestational age Pediatr Neonatol 2011 52 219 222 10.1016/j.pedneo.2011.05.012 21835368

[B27] de Jong EM de Haan MA Gischler SJ Hop W Cohen-Overbeek TE Bax NM de Klein A Tibboel D Grijseels EW Pre- and postnatal diagnosis and outcome of fetuses and neonates with esophageal atresia and tracheoesophageal fistula Prenat Diagn 2010 30 274 279 20112230 10.1002/pd.2466

[B28] Dolk H Loane M Garne E The prevalence of congenital anomalies in Europe Adv Exp Med Biol 2010 686 349 364 10.1007/978-90-481-9485-8_20 20824455

[B29] Dolk H Loane M Garne E Congenital heart defects in Europe: prevalence and perinatal mortality, 2000 to 2005 Circulation 2011 123 841 849 10.1161/CIRCULATIONAHA.110.958405 21321151

[B30] Henrich K Huemmer HP Reingruber B Weber PG Gastroschisis and omphalocele: treatments and long-term outcomes Pediatr Surg Int 2008 24 167 173 10.1007/s00383-007-2055-y 17985136

[B31] Holland AJ Walker K Badawi N Gastroschisis: an update Pediatr Surg Int 2010 26 871 878 10.1007/s00383-010-2679-1 20686898

[B32] Kotecha S Barbato A Bush A Claus F Davenport M Delacourt C Deprest J Eber E Frenckner B Greenough A Nicholson A Nton-Pacheco JL Midulla F European respiratory society task force on congenital diaphragmatic hernia Eur Respir J 2011 39 820 829 22034651 10.1183/09031936.00066511

[B33] United Nations Population Fund Report of the International Workshop on Skewed Sex Ratios at Birth: Addressing the Issue and the Way Forward 2011 Hanoi

[B34] Samms-Vaughan ME Ashley DC Caw-Binns AM Factors determining admission to neonatal units in Jamaica Paediatr Perinat Epidemiol 2001 15 100 105 10.1046/j.1365-3016.2001.00332.x 11383573

[B35] Mukhtar-Yola M Iliyasu Z A review of neonatal morbidity and mortality in Aminu Kano Teaching Hospital, northern Nigeria Trop Doct 2007 37 130 132 10.1258/004947507781524683 17716492

[B36] Mmbaga BT Lie RT Kibiki GS Olomi R Kvale G Daltveit AK Transfer of newborns to neonatal care unit: a registry based study in Northern Tanzania BMC Pregnancy Childbirth 2011 11 68 10.1186/1471-2393-11-68 21970789 PMC3206461

[B37] Neogi SB Malhotra S Zodpey S Mohan P Assessment of special care newborn units in India J Health Popul Nutr 2011 29 500 509 22106756 10.3329/jhpn.v29i5.8904 PMC3225112

[B38] Ministry of Health of Viet Nam National Guidelines for Reproductive Health 2009 Hanoi: Care Services (circulated under the decision No 4620/QD-BYT)

